# The impact of mental distress on influenza vaccine coverage

**DOI:** 10.1371/journal.pone.0266692

**Published:** 2022-04-07

**Authors:** Linda Hassouneh, Shira Dunsiger

**Affiliations:** 1 Division of Infectious Diseases, Department of Pediatrics, Warren Alpert Medical School of Brown University, Providence, Rhode Island, United States of America; 2 Center for Health Promotion and Health Equity, School of Public Health, Brown University, Providence, Rhode Island, United States of America; Konkuk University, REPUBLIC OF KOREA

## Abstract

Influenza is a major cause of morbidity and mortality worldwide. The flu vaccine is the most important strategy to prevent influenza. Studies indicate that individuals with mental health disorders are at an increased risk of comorbid health conditions that predispose them to severe flu complications. This study examined the association between mental distress and influenza vaccine coverage among non-institutionalized adults in the United States. Data was analyzed from the 2016 Behavioral Risk Factor Surveillance System (BRFSS). The analytic sample (453,924) included those with valid information on health-related quality of life (HRQOL) and flu vaccine coverage. Bivariate analysis and logistic regression were performed. Those with infrequent mental distress had 1% (95% confidence interval [CI] 0.96,1.03) lower odds and those with frequent mental distress had 21% (95% CI 0.75,0.82) decrease odds of receiving the flu shot in comparison to those with no mental distress, given all else equal. A negative effect on influenza vaccination rates was observed with frequent mental distress when compared to those with no mental distress. Further studies are warranted to better understand this association.

## Introduction

Vaccines are one of the most successful measures in improving health worldwide, however vaccination rates continue to be suboptimal and have prompted global initiatives to improve coverage [1–5]. Influenza, a vaccine preventable illness, is a highly contagious, acute respiratory illness that affects all age groups [6,7]. Per the Centers for Disease Control and Prevention (CDC), influenza caused about 9–41 million illnesses, 140,000–710,000 hospitalizations and 12,000–52,000 deaths per year between 2010 and 2020 [CDC, 8]. The influenza vaccine is a crucial step in preventing the flu and reducing disease burden [9,10, CDC, 11]. It was estimated that the influenza vaccine prevented 5.3 million illnesses, 2.7 million medical visits, 70,000 hospitalizations and 6,400 influenza related deaths during the 2015–2016 season [CDC, 12]. In the United States (US), vaccine coverage among adults 18 years and older averaged 37.1–50.2% over the past 10 years, the lowest being influenza season 2017–2018 and the highest recorded during 2020–2021 season [CDC, 8]. The increase in vaccination coverage over the past few seasons is encouraging but is still not sufficient as the Healthy People 2030 goal is to vaccinate 70% of all persons aged 6 months and over against seasonal influenza [1].

In recent years, research has highlighted the health disparities among individuals with mental health disorders [13]. Mental health illnesses are associated with an increase in comorbid medical conditions and mortality in comparison to the general population [14–18]. These comorbidities, such as cardiovascular disease, diabetes mellitus and human immunodeficiency virus (HIV)/acquired immunodeficiency syndrome (AIDS), are known risk factors for serious flu related complications [18, CDC, 19]. Studies have found that persons with mental health disorders were less likely to receive preventive health services, such as cancer screening and vaccinations [20–26]. In these studies, mental health disorders were associated with a lower likelihood of receiving influenza vaccination, but the populations assessed were not generalizable and pertained to specific groups, such as elderly, women, homeless or individuals with major depression.

Overall, the literature evaluating the impact of mental distress on vaccination coverage is scant. The effects of mental health on influenza vaccine coverage among the average adult population with mental distress with no formal psychiatric diagnosis is unknown. Given the ongoing threat of influenza and other vaccine preventable diseases, such as Coronavirus Disease 2019 (COVID-19), on global health it is crucial to identify groups of individuals that are more likely to be unvaccinated and identify the disparities in our health system to better improve public health.

The purpose of this study was to examine the association between mental distress and influenza vaccine coverage among a large, national database of noninstitutionalized US adults. It is hypothesized that frequent mental distress will be negatively associated with influenza vaccination rates.

## Methods

For the current study, data was analyzed from the 2016 Behavioral Risk Factor Surveillance System (BRFSS) [27]. The BRFSS is an annual health-related telephone survey supported by the CDC that aims to collect health-related risk behaviors, chronic health conditions and use of preventive services from noninstitutionalized adult (18 years and older) population living in the United States [CDC, 28]. 486,302 adults were surveyed in the 2016 BRFSS. The analytic sample (453,924) included those with valid information on health-related quality of life and flu vaccine coverage.

Mental health was the exposure of interest and assessed in the BRFSS as part of the health-related quality of life (HRQOL) section. Mental health was self-reported based on participants’ response to the question “Now thinking about your mental health, which includes stress, depression, and problems with emotions, for how many days during the past 30 days was your mental health not good?” For the purpose of this analysis, data was further categorized into three groups: those who reported 0 days were categorized as having no mental distress and those who reported 1–13 days were categorized as having infrequent mental distress. As used in earlier studies frequent mental distress was assigned to those who responded 14 days or greater [29–31].

The outcome variable was flu vaccine coverage and analyzed as a dichotomous variable: received or not received. Vaccine coverage was specific only to the past year with the question “During the past 12 months, have you had either a flu shot or a flu vaccine that was sprayed in your nose:” For both exposure and outcome variables, those that refused or responded don’t know/not sure were considered invalid data, along with those with missing responses. Only 6.7% of the eligible population (5% flu vaccine, 1% HRQOL) was excluded from analytic sample.

Potential confounders included demographic variables such as age, race and gender. Age was categorized into 3 groups: 18–34 years, 35–54 years and greater than 55 years of age. Ethnicity was separated into 5 groups: white, black, Hispanic, Asian and other. Other included multiracial, Native Hawaiian or other Pacific Islander, American Indian or Alaskan Native and those that responded “other race.” Health insurance was included in the analysis as those without insurance may have fewer medical resources available. Employment was operationalized as a binary indicator of current employment. Employees are required to receive the annual influenza shot by certain institutions and therefore was important to add into the analysis. A respondent was considered employed if they were employed for wages or self-employed and unemployed if they were out of work, a homemaker, a student, retired or unable to work. An individual may be encouraged to receive the flu shot if they have children in the household and this was also considered a possible confounder. Those that gave a number besides 0 as to how many children less than 18 years of age live in the household were considered to have children in their household. Depressive disorder is a psychiatric illness that may highly correlate with mental distress and therefore included as a covariate. Those that answered yes to ever being told that they have a depressive disorder, including depression, major depression, dysthymia, or minor depression were considered to have a depressive disorder. Lastly, it is well known that individuals with chronic lung disease are at increased risk of developing severe complications to influenza and are strongly encouraged to receive the flu shot [CDC, 19]. Chronic obstructive pulmonary disease (COPD) and asthma are two chronic lung disorders that were included as covariates. For all variables, individuals that refused or responded don’t know/not sure were analyzed as invalid data, along with the missing. Invalid data/missing was excluded from the bivariate analysis and Table 1 as it accounted for <5% of observations.

**Table 1:** Characteristics of US adults categorized into status of mental health, BRFSS 2016.

	No mental distress 311,871 (65.5%)	Infrequent mental distress 93,685 (22.8%)	Frequent mental distress 48,368 (11.7%)	*P* value
**Flu vaccine**	** **	** **	** **	*P* < .001
Yes	144,277 (39.5%)	40,663 (37.1%)	204,587 (38.4%)	
No	167,594 (60.5%)	53,022 (62.9%)	28,721 (65.5%)	
**Sex**	** **			*P* < .001
Male	144,977 (52.3%)	34,021 (42.2%)	17,179 (40.1%)	
Female	166,868 (47.7%)	59,656 (57.8%)	31,176 (59.9%)	
**Age**	** **			*P* < .001
18–34 years	38,566 (25.7%)	21,264 (40.4%)	9,436 (34.7%)	
35–54 years	75,448 (32.1%)	29,701 (33.8%)	15,686 (35%)	
> = 55 years	193,576 (42.2%)	41,955 (25.7%)	22,828 (30.3%)	
**Race**	** **			*P* < .001
White	239,440 (63.6%)	72,010 (65.5%)	35,339 (65.2%)	
Black only	24,365 (11.5%)	7,380 (11.4%)	4,190 (12.3%)	
Hispanic	24,440 (17.1%)	6,968 (14.7%)	4,233 (15.1%)	
Asian	6,977 (5.36%)	1,928 (5.21%)	575 (2.66%)	
Other	11,447 (2.53%)	4,168 (3.11%)	3,091 (4.79%)	
**Health insurance**	** **			*P* < .001
Yes	291,031 (89.3%)	86,487 (89%)	42,999 (85.7%)	
No	19,809 (10.7%)	6,909 (11%)	5,160 (14.3%)	
**Employment**	** **			*P* < .001
Employed for wages	153,637 (58.5%)	51,501 (60.2%)	17,825 (43.4%)	
Un-employed	156,149 (41.5%)	41,718 (39.8%)	30,231 (56.6%)	
**Children in household**	** **			*P* < .001
Yes	72,242 (34.6%)	29,006 (39%)	13,754 (36.9%)	
No	238,327 (65.4%)	64,337 (61%)	34,427 (63.1%)	
**Depressive disorder**	** **			*P* < .001
Yes	23,999 (6.7%)	27,012 (25.5%)	28,654 (56.3%)	
No	287,210 (93.3%)	66,191 (74.5%)	19,304 (43.7%)	
**Asthma**	** **			*P* < .001
Yes	34,236 (10.8%)	15,578 (16.6%)	11,581 (24.2%)	
No	276,919 (89.2%)	77,833 (83.4%)	36,550 (75.8%)	
**COPD**				*P* < .001
Yes	20,772 (4.95%)	8,072 (6.51%)	9,048 (15.2%)	
No	290,003 (95.1%)	85,210 (93.5%)	38,938 (84.8%)	

Note: Observations excluded from the table as percent invalid/missing was <5.

### Ethics statement

BRFSS data is publicly available with no identifiable private information; therefore, Institutional Review Board (IRB) approval was not required [32].

### Statistical analysis

All statistical analysis was performed using StataSE version 15 [33]. The data was weighted to adjust for non-response, non-coverage, imbalance in the selected sample and to allow projects from the sample to the general population. In addition, analyses accounted for complex survey design to obtain correct standard errors. Bivariate analyses of outcome variable and covariates (demographics, medical comorbidities) among those with mental distress was conducted to define characteristics of the study population. Chi square tests were used to examine associations between exposed and non-exposed groups with respect to categorical demographic variables. All variables were tested individually for inclusion into a final adjusted model. If the difference between the crude and adjusted odds ratio (OR) for each variable was greater than 10% the variable was included in the final adjusted mode. Health insurance and employment were included in the final adjusted model although the difference was <10% due to the potential strong association between these variables and vaccination coverage. Logistic regression analysis was conducted to compare influenza vaccination and mental distress, adjusting for race, depressive disorder, health insurance status and employment.

## Results

The majority of the study population (65.5%) had no mental distress. 22.8% reported infrequent mental distress and 11.7% were categorized as having frequent mental distress. 39.5% of those with no mental distress received the flu shot. 37.1% of individuals with infrequent mental distress received the flu shot and 38.4% of those with frequent mental distress were vaccinated. Males accounted for 52.3% of those with no mental distress, 42.2% of respondents with infrequent mental distress and 40.1% in the group with frequent mental distress. The largest percentage (42.2%) of individuals with no mental distress at least 55 years of age. Among those with infrequent mental distress, the largest percentage (40.4%) were 18–34 years of age. 35% of those with frequent mental distress were 35–54 years of age. Regarding race, most were white followed by Hispanics. 85–89% of the study population had health insurance. Individuals with infrequent mental distress were more likely to be employed (60.2%) in comparison to 43.4% of those with frequent mental distress and 58.5% of those with no mental distress. Not having children was seen in 61% and 63.1% with infrequent mental distress and frequent mental distress respectively. 65.4% of individuals with no mental distress did not have children in their household. Most individuals (56.3%) with frequent mental distress also had a depressive disorder, in comparison to 6.7% and 25.5% of those with no mental and infrequent mental distress respectively. Most of the study population did not have asthma and COPD. A higher percentage (24.2 and 15.2%) were diagnosed with asthma and COPD respectively in individuals with frequent mental distress (all *P* < .001) [Table 1].

Results indicate that mental distress was significantly associated with influenza vaccination coverage. Specifically, those with frequent mental distress had 19% lower odds (OR = 0.81, 95% confidence interval [CI] 0.78–0.84) of receiving the flu shot compared to those without mental distress. Females were 32% (OR = 1.32, 95% CI 1.29–1.35) more likely to receive the influenza vaccine in comparison to males. Those aged 55 years and older had 2.61 (95% CI 2.53–2.70) the odds of becoming vaccinated in comparison to those aged 18–34 years. Hispanics, blacks, and all other racial groups were less likely to receive the flu shot in comparison to whites. Those without medical insurance had 68% (OR = 0.32, 95% CI 0.30–0.33) lower odds of becoming vaccinated in comparison to adults that were insured. Unemployed individuals had 56% (OR = 1.56, 95% CI 1.52–1.60) increase odds of receiving the flu shot in comparison to those who were employed for wages. Those who lived in households with children were 39% (OR = 1.39, 95% CI 1.36–1.43) more likely to receive the flu shot in comparison to households without children. Those who were ever told that they had a depressive disorder had an 18% (OR = 1.18, 95% CI 1.14–1.22) increase odds of receiving the flu shot in comparison to those without a depressive disorder. Individuals without asthma and COPD were 18% (OR = 0.82, 95% CI 0.80–0.85) and 40% (OR = 0.60, 95% CI 0.58–0.63) less likely to receive the influenza vaccine shot in comparison to patients with asthma and COPD respectively [Table 2].

**Table 2:** Unadjusted and adjusted OR of US adults having received the influenza vaccine among US adults, BRFSS 2016.

	Unadjusted OR (95% CI)	Adjusted OR (95% CI)
**Mental health**	** **	** **
No mental distress	1.00	1.00
Infrequent mental distress	0.90 (0.88–0.93)	0.99 (0.96–1.03)
Frequent mental distress	0.81 (0.78–0.84)	0.79 (0.75–0.82)
**Sex**	** **	
Male	1.00	
Female	1.32 (1.29–1.35)	
**Age**	** **	
18–34 years	1.00	1.00
35–54 years	1.18 (1.13–1.22)	1.16 (1.12–1.21)
> = 55 years	2.61 (2.53–2.70)	2.22 (2.15–2.31)
**Race**	** **	
White	1.00	
Black only	0.68 (0.65–0.71)	
Hispanic	0.61 (0.59–0.64)	
Asian	1.09 (1.00–1.18)	
Other	0.77 (0.71–0.82)	
**Health insurance**	** **	
Yes	1.00	1.00
No	0.32 (0.30–0.33)	0.38 (0.36–0.40)
**Employment**	** **	
Employed for wages	1.00	1.00
Un-employed	1.56 (1.52–1.60)	1.22 (1.18–1.25)
**Children in household**	** **	
Yes	1.00	
No	1.39 (1.36–1.43)	
**Depressive Disorder**	** **	
Yes	1.18 (1.14–1.22)	1.25 (1.21–1.30)
No	1.00	1.00
**Asthma**	** **	
Yes	1.00	
No	0.82 (0.80–0.85)	
**COPD**		
Yes	1.00	
No	0.60 (0.58–0.63)	

**Fig 1:**
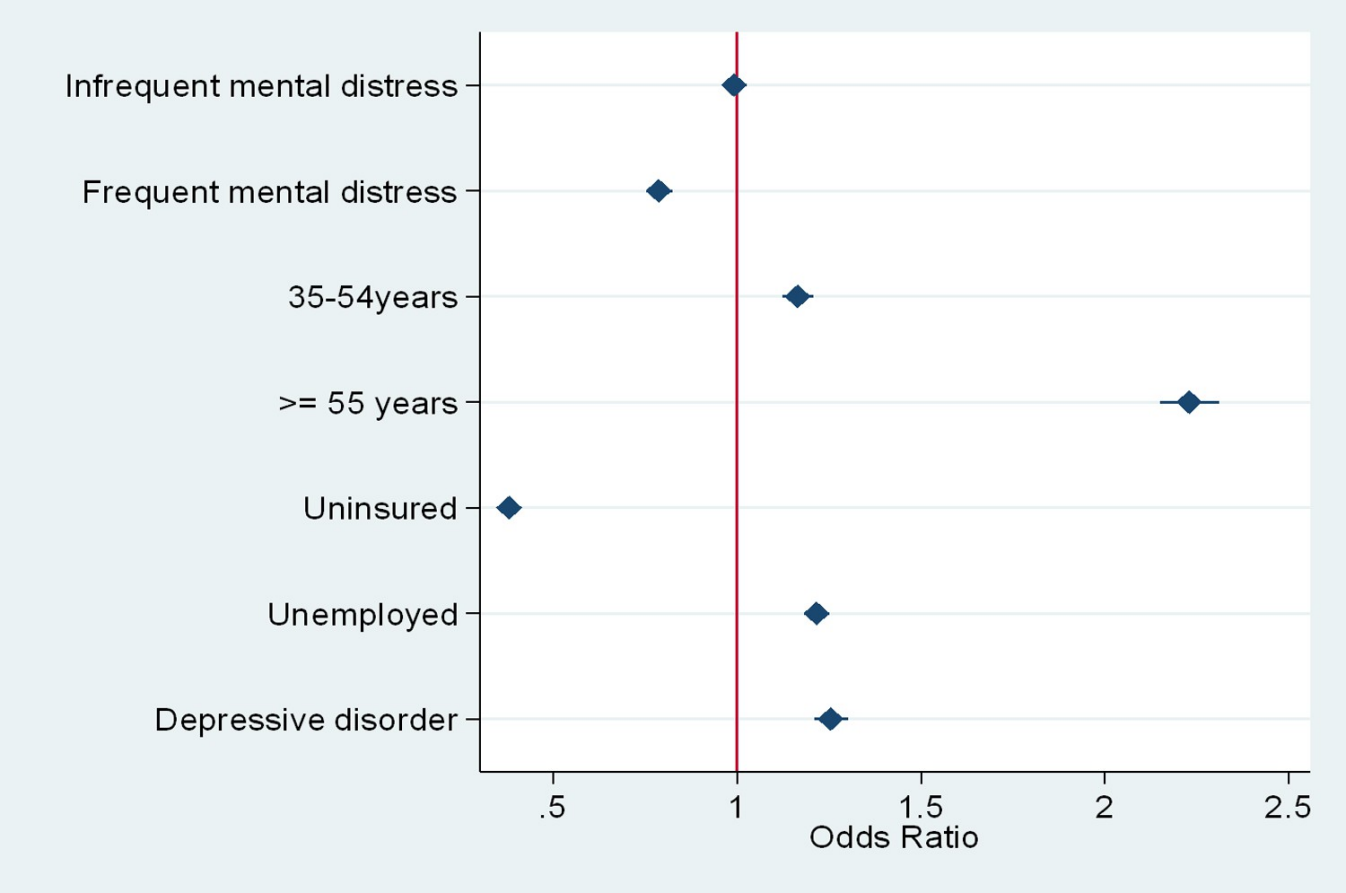
Forest plot of odds ratio for outcome variable.

An adjusted OR was estimated using logistic regression with the following covariates: age, insurance, employment, and depressive disorder. The adjusted model shows a strong association between frequent mental distress and flu vaccination, but no statistically significant association related to infrequent mental distress (Fig 1). Persons with frequent mental distress had 21% (95% CI 0.75,0.82) lower odds and those with infrequent mental distress had 1% (95% CI 0.96,1.03) lower odds of receiving the flu shot in comparison to those with no mental distress, given all else equal. Those aged 55 years and older had 2.22 (95% CI 2.15, 2.31) the odds of receiving the flu shot in comparison to those aged 18–34 years, given all else equal. Medically uninsured adults were 62% (95% CI 0.36, 0.40) less likely to receive the flu shot in comparison to those who were insured. Those with depressive disorder were 25% (95% CI 1.21, 1.30) more likely to receive the flu shot in comparison to those without a depressive disorder, given all else equal [Table 2].

## Discussion

In brief, this study showed an association between mental distress and influenza vaccination. This is similar to what has been shown in the literature, namely, that those with mental illness had a lower likelihood of receiving flu vaccination [20–22,25,26,34]. We found that depressive disorder was associated with an increased odds of receiving the flu shot in comparison to those without a depressive disorder, after adjusting for confounders. This may suggest that those with formal diagnoses are more frequently under physician care which might increase the likelihood of receiving information about vaccination and ultimately receipt of vaccines such as the influenza vaccine. Further studies could perhaps investigate the access to vaccine services among those with co-existing conditions including mental health disorders. Although the current study supported other published literature on the topic, some findings were contradictory to published work. For example, Druss et al observed that individuals with untreated depression were more likely to have not received influenza vaccination [24]. The current study did not include information on access to treatment for depressive disorders.

We were unable to fully identify the barriers to vaccination of the study population, but analyses suggests that health insurance was associated with a higher likelihood of receiving the vaccine. In the US, health disparities among racial and ethnic groups are well established. In this study, certain racial groups, such as blacks, Hispanics, Native Hawaiian or other Pacific Islander and American Indian or Alaskan Native, were associated with lower odds of receiving flu vaccination in comparison to whites. This emphasizes the continued need to advance health equity in the United States.

With the novel coronavirus disease 2019 (COVID-19), the psychological impact of the pandemic is becoming more apparent and reported in the literature [35–39]. Mental health issues are occurring due to many factors, including infection with the severe acute respiratory syndrome coronavirus 2 (SARS CoV-2) virus, heavy workloads, unemployment, lockdowns, and quarantines. Those with pre-existing mental health disorders are more vulnerable to the psychological impact of the pandemic [36]. Although this is outside the scope of this study, one could imagine from our results that perhaps mental distress brought on by the pandemic could affect not only influenza vaccination but also COVID-19 vaccination. It is important for primary health care workers to screen patients for mental health problems and provide the appropriate health care, including preventive health services.

This study has several limitations. First, all data was analyzed from self-reported responses that may lead to recall bias. There was no medical data available for comparison to validate answers. Second, the study population was large but did not include homeless individuals, which is an important population to include when assessing for mental distress. Whether these individuals are also unvaccinated against influenza warrants further investigations. Lastly, mental health was evaluated over the past 30 days, while flu vaccine was over the past 12 months. Therefore, the BRFSS survey only captured the mental health status of the individual one month prior to the interview and this may or may not correlate with the person’s mental health status over the previous year.

## Conclusion

A negative effect on influenza vaccination rates was observed with frequent mental distress when compared to those with no mental distress. Physicians may need to screen patients for mental distress and ensure those with mental distress are provided adequate preventive healthcare and attention.
